# MEMS Underwater Directional Acoustic Sensor in Near Neutral Buoyancy Configuration

**DOI:** 10.3390/s22041337

**Published:** 2022-02-10

**Authors:** Fabio Alves, Jaehyun Park, Leland McCarty, Renato Rabelo, Gamani Karunasiri

**Affiliations:** Naval Postgraduate School, Monterey, CA 93943, USA; jaebbong85@gmail.com (J.P.); leland.mccarty@gmail.com (L.M.); rcrabelo.br@nps.edu (R.R.); gkarunas@nps.edu (G.K.)

**Keywords:** neutral buoyancy, MEMS acoustic sensor, directional acoustic sensor, underwater

## Abstract

A MEMS directional acoustic sensor housed in an air cavity and operated underwater in a near-neutral buoyancy configuration is demonstrated. The sensor consists of two wings connected by a bridge and attached to a substrate by two centrally mounted torsional legs. The frequency response showed two resonant peaks corresponding to a rocking mode (wings moving in opposite directions) and a bending mode (wings moving in the same direction). Initial tests of the sensor using a shaker table showed that the response is highly dependent on the vibration direction. In air, the sensor showed a maximum sensitivity of about 95 mV/Pa with a cosine directional response. Underwater, the maximum sensitivity was about 37 mV/Pa with a similar cosine directional response. The measured maximum SNR was about 38 dB for a signal generated by a sound stimulus of 1 Pa when the sensor is operated near the bending resonance. The results indicate that this type of MEMS sensor can be operated in a near-neutral buoyant configuration and achieve a good directional response.

## 1. Introduction

Detection of underwater sound in a neutral buoyancy configuration is typically achieved by sensing the motion of the entire assembly that houses the sensor. When a sound is transmitted through a medium such as water, the medium vibrates in the sound propagation direction. At neutral buoyancy the enclosure has the same mass as the water it replaces making it respond to sound in the same way as the replaced volume of water [1,2,3,4].

Sensing the motion of the medium has been studied for decades by various research groups [1,2,3,4,5,6,7,8,9,10,11,12,13,14,15,16,17,18,19,20,21,22,23]. Leslie et al. [1] described the operation of hydrophones enclosed within a spherical housing. They reported a flat sensor response from 15 to 700 Hz, with a sensitivity of 0.18 V/(cm/s). These authors also concluded that the directional response of the velocity sensor is better at low frequencies than high frequencies, proving over −20 dB of cross-axial sensitivity. The researchers attributed this finding to the lack of mechanical rigidity of the hollow brass sphere at higher frequencies, resulting in a substantial transverse response [1]. A different approach for designing a sensor with an intrinsic directional response is shown by the polyvinylidene fluoride (PVF2) biomorph studied by Josserand and Maerfeld [15]. Their findings showed that a flexible PVF2 biomorph’s piezoelectric properties allowed for a dipole directional response at 60 Hz, with a 25 dB signal level difference between sound incident at 0° and 90°. The biomorph’s sensitivity was −190 dB re 1 V/μPa from 1 to 4 kHz, although it was not completely flat [15].

A micro-electro-mechanical system (MEMS) based direction-finding inertial sensor was demonstrated by Rockstad et al., using an electron-tunneling transduction mechanism [16]. The micromachined sensor was made up of a hinged, cantilevered proof mass with a tunneling tip. To sense proof mass vibrations on its cantilever, the edge of the proof mass contains an electrode that interacts with a second cantilever tunneling electrode. Their research goal was to develop and test a miniature directional sensor with a self-noise below 100 ng/√Hz from 5 Hz to 1 kHz [16]. Shaker table laboratory testing of a single-axis electron tunneling sensor showed a noise floor from 1 μg to 10 ng/√Hz. Results in a neutrally buoyant configuration were not published by the authors.

Roh et al. [17] demonstrated a shear type accelerometer, used in conjunction with hydrophones, to use for localization and characterization of acoustic sources. Their efforts focused on the detailed structural design of the accelerometer to optimize the vector sensor system. They reported a cross-axial sensitivity of −20 dBV, and when the accelerometer was paired with an omnidirectional hydrophone, a cardioid polar response was produced. Their accelerometer also proved to have a receiving voltage sensitivity of −204.9 dB (re 1 V/μPa).

Zhang et al. [5] studied an integrated circuit piezoelectric accelerometer encapsulated in a polyurethane, possibly neutrally buoyant, cylinder, that was designed to move freely along with the sound wave. The researchers reported a sensitivity of −185 dB re 1 V/μPa at 100 Hz, and unstable results beyond 1 kHz.

The accelerometer studied by Zhang et al. measured 20 mm by 20 mm, and required a large, co-vibration, elastic mounting system measuring 43 mm by 127 mm. The sensitivity measured on a shaker table was approximately 7.8 V/g [5]. The authors employed a rigid cylinder to assemble a vector sensor, and indicated that when the sound wavelength is much greater than the cylinder size, the cylinder moves freely with the sound wave. For underwater directional response measurement, sensor housings are typically fixed onto a frame using a set of low-stiffness springs to lower their resonance frequency below 1 Hz [4,6].

Of particular interest is the possibility of employing a single MEMS sensor, with simple readout electronics, to achieve directionality and a high signal-to-noise ratio in a neutrally buoyant configuration. One attractive way to achieve this is to design a MEMS sensor with resonance in the middle of the frequency range of interest, privileging a narrow band operation around it. In this paper, fabrication and characterization of MEMS directional acoustic sensors designed to operate in neutrally buoyant configuration are described. Since the MEMS sound sensor employed in this work has an operating frequency much higher than 1 Hz, the effects of suspension on the sensor response are negligible [5].

There have been some studies of MEMS-based underwater sensors. Guan et al. [18] reported on a T-shaped vector sensor that uses a pair of long cantilever beams with piezoresistors on its transverse sections. Deformation of the beams caused by an underwater acoustic signal induces a resistance variation that is related to the direction and pressure of the wave. The authors report a sensitivity between −181 dB and −170 dB at 1 kHz and an 8-shape directional pattern. Xue et al. [19] demonstrated a bionic vector sensor based on a solitary vertical cylinder that rests in the center of a four-orthogonal beam structure. Acoustic waves incident to the solitary vertical cylinder create compressive and tensile stresses in the structure. These stresses are transduced to voltage by the piezoresistive effect of resonant tunneling diodes. A sensitivity of −184.6 dB at 1 kHz and a dipole directional pattern is reported. Edalatfar et al. [20] demonstrated a MEMS accelerometer micromachined from a silicon-on-insulator (SOI) wafer with “interdigitated capacitive comb fingers for electronic readout.” This sensor was operated in a neutrally buoyant configuration to measure particle velocity of the water. Sensitivity up to −208 dB at 500 Hz, and a dipole directivity pattern, were achieved. Common characteristics among these MEMS sensors are complexity in fabrication and low sensitivity. The *Ormia*-inspired MEMS based acoustic sensor reported in this paper is relatively simple to fabricate and exhibits a dipole directional response and significantly higher sensitivity.

## 2. Materials and Methods

**Figure 1:**
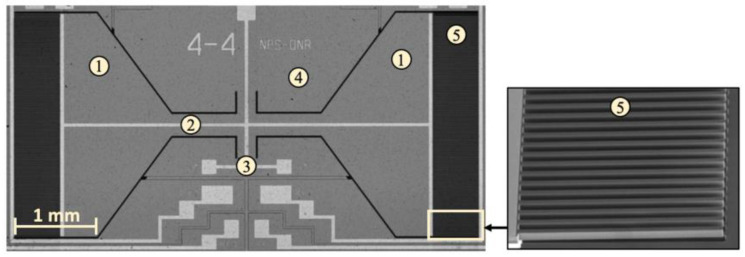
Optical micrograph of the MEMS sensor with comb finger capacitors attached to the edges of the wings. (1) Wings; (2) bridge; (3) leg; (4) substrate; and (5) interdigitated comb fingers.

The MEMS acoustic sensor designed by our group is based on the binaural hearing principle of the *Ormia ochracea* parasitic fly. A detailed study of the *Ormia*’s hearing organ and its mechanical equivalent can be found in [21]. It consists of two 6.6 mm^2^ trapezoidal wings connected in the middle by a 2 mm long, 300 μm wide bridge. This structure is connected to a Si substrate using two 120 μm long, 80 μm wide torsional legs as shown in Figure 1.

**Figure 2:**
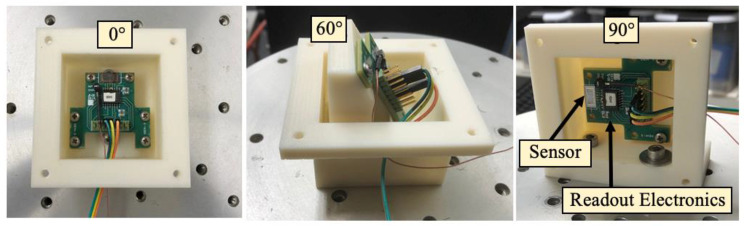
Sensor and electronic readout printed circuit board mounted on the shaker at 0, 60, and 90 degrees.

The thickness of the mechanical structure of the sensor is 25 μm, whereas the substrate is 400 μm thick. A trench on the back side assures that the sensor is free to oscillate. The sensor was designed using finite element modeling (COMSOL Multiphysics) and fabricated by MEMSCAP, a commercial foundry specializing in the Silicon-on-Insulator Multi-User Manufacturing Process (SOIMUMPS). The displacement of the sensor’s wings is transduced to a voltage using 500 μm long, 10 μm wide interdigitated comb-finger capacitors attached to the edges of the wings and the substrate. The gap between fingers attached to the wings and substrate is 2.5 μm, which provided a total capacitance of about 6 pF for each wing (measured using a semiconductor parametric analyzer). The sensor has two dominant resonant modes: bending and rocking. In the bending mode, the wings move in the same direction; in the rocking mode the wings move in opposite directions [24]. A Micro Sensors MS 3110 capacitive readout IC [25] is used to convert wing movement to an output voltage. Before testing the sensor in the neutral buoyancy configuration, preliminary tests were conducted using a shaker table in a quiet room where no acoustic stimulation was provided. To vibrate the sensor at different angles, five fixtures were fabricated to mount the circuit board containing the sensor tilted at: 0 degrees, 30 degrees, 45 degrees, 60 degrees, and 90 degrees (Figure 2). Accelerations were measured using a calibrated Endevco^®^ model 22 Picomin™ accelerometer.

**Figure 3:**
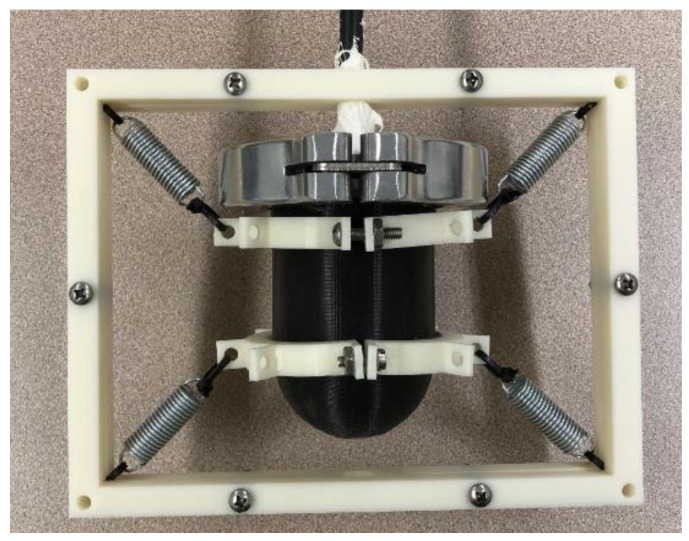
Sensor housing (black) mounted to a frame (white) using a set of low stiffness springs for underwater testing. The frame provides stability during directional response measurements.

To operate the MEMS sensor in the near-neutral buoyancy configuration, a watertight housing was fabricated using EpoxAcast^TM^ 690 epoxy resin as shown in Figure 3. The dimensions of the rigid housing are 50.8 mm inner diameter, 3 mm thickness, and 80 mm length. To prevent the sensor housing from rotating when underwater and to improve direction control during the measurements, a rectangular frame with low-stiffness anchoring springs was employed. The dimensions of the frame are 195 mm in length and 150 mm in height.

Figure 3 shows a photograph of the sensor assembly mounted on the frame. The measured transmission coefficient of the housing material was found to be about 6 × 10^−4^ indicating that hardly any sound would transmit through it. This indicates that the sensor response is primarily due to vibrational motion of the entire housing when it interacts with the incident sound waves.

The MEMS sensor was characterized in air in an anechoic chamber. To compare the responses of the sensor at various stages of the assembly, frequency response measurements were performed without the housing, with the housing, and with the housing and frame. In each configuration, the sensor assembly and a reference microphone were mounted on a turntable pole for directional response measurements. The calibrated reference microphone was used to measure the sound pressure near the sensor assembly. The frequency responses for all three configurations were measured at normal incidence from 1 to 2 kHz. The sensitivity (V/Pa) was obtained by dividing the voltage output of the sensor by the sound pressure measured by the reference microphone.

Finally, underwater measurements of the assembled sensor were performed in a water tank to determine frequency and directional responses. The sensor assembly and a sound projector were suspended at a 1-m depth in the water tank. The distance between the sensor and the projector was 0.75 m. A calibrated B&K 8103 hydrophone was utilized to measure the incident sound pressure at the location of the sensor. The frequency response measurement was conducted from 1 to 2 kHz for the MEMS sensor and the hydrophone separately.

## 3. Results

### 3.1. Acceleration Measurements

**Figure 4:**
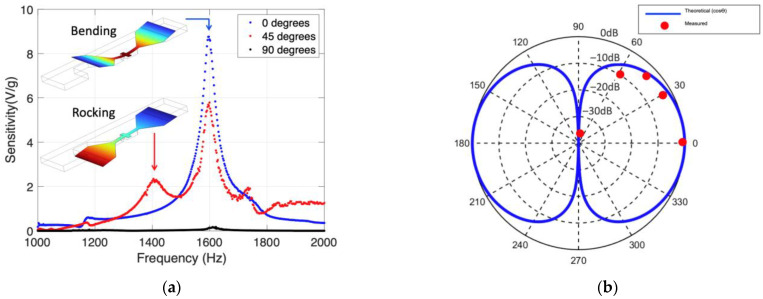
(**a**) Frequency-dependent sensitivity (V/g) of the MEMS sensor mounted on a shaker at 3 different angles. The insets are FE simulations of the bending and rocking vibrational modes at 1600 and 1440 kHz, respectively. (**b**) Directional response at resonance at 5 different vibration angles (circular markers) along with the cosine of the vibration angle (solid line). The magnitude scale in the polar plot represents the measured voltage normalized by the maximum value obtained at zero degrees.

The frequency-dependent sensitivity (V/g) of the MEMS sensor is shown in Figure 4a for three different vibration directions. The measurements clearly show a resonance peak at 1.6 kHz due to the sensor’s resonant bending mode. As the tilt angle is increased, the sensitivity is reduced, making the sensor response highly directional with respect to the shaker vibration direction. The minute response at resonance when the sensor is vertically positioned (Figure 4a, solid blue line) is most likely due to mechanical coupling of the mount and a small in-plane movement of the shaker. In addition, at 45 degrees an additional peak appears near 1.44 kHz, which corresponds to the rocking motion of the two wings. The vibrational modes were obtained in COMSOL (insets of Figure 4a) confirming that 1.6 kHz is the bending mode and 1.44 kHz is the rocking vibrational mode. In addition, the absence of the peak at 1.44 kHz at normal incidence (0°) indicates that this peak is due to the rocking motion, since this mode is not excited when the sound pressure is perpendicular to the sensor. Similarly, the appearance of the peak at 1.6 kHz at normal incidence indicates that this peak is due to the bending mode since both wings can bend symmetrically due to the full force of the sound pressure. The additional feature around 1.7 kHz is most likely due to a resonance induced by the mounting hardware. The directional response of the sensor shows a cosine dependence with respect to the vibration direction, similar to the directional response of MEMS sensors stimulated acoustically [24]. Figure 4b shows the sensor response at 5 different vibration angles (red dots) and a theoretically predicted cosine (solid line) for comparison. The rotational measurements were performed at the resonant frequency of the sensor.

### 3.2. Anechoic Chamber Measurements

**Figure 5:**
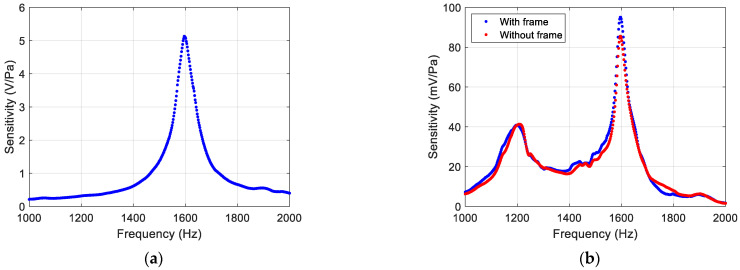
Measured frequency response of the sensor at normal incidence: (**a**) without the housing and (**b**) with the housing and frame (blue dots), and with the housing only (red dots).

Figure 5a shows the sensitivity as a function of frequency for the bare sensor, showing a resonance peak at around 1.6 kHz with sensitivity of about 5 V/Pa and a quality factor of 24. Off-resonance, the average sensitivity was found to be 100 mV/Pa whereas the directional characteristics are maintained. The origin of the peak is the bending motion of the two wings, which predominates at the normal incidence of sound [12]. Figure 5b shows the sensitivity for the other two configurations, which show a drastic reduction of sensitivity (95 mV/Pa) compared to that without the housing (5 V/Pa).

The peak sensitivity of the sensor with the housing is only about 2% of the sensitivity of the bare sensor. However, based on the measured transmissivity of the housing material (~ 6 × 10^−4^), the expected response should have been about 0.06%. Thus, the observed 2% is about 30 times higher than the predicted value. This indicates that the sensor response with the housing is predominantly due to the motion of the entire assembly. Figure 5b also indicates that the presence of the frame and the springs does not adversely affect the sensitivity of the sensor. The measured frequency responses of the sensor shown in Figure 5b exhibit an additional peak near 1.2 kHz, which was not present in the frequency response of the bare sensor (Figure 5a). This peak is most likely due to an excitation mode introduced by the sensor housing.

**Figure 6:**
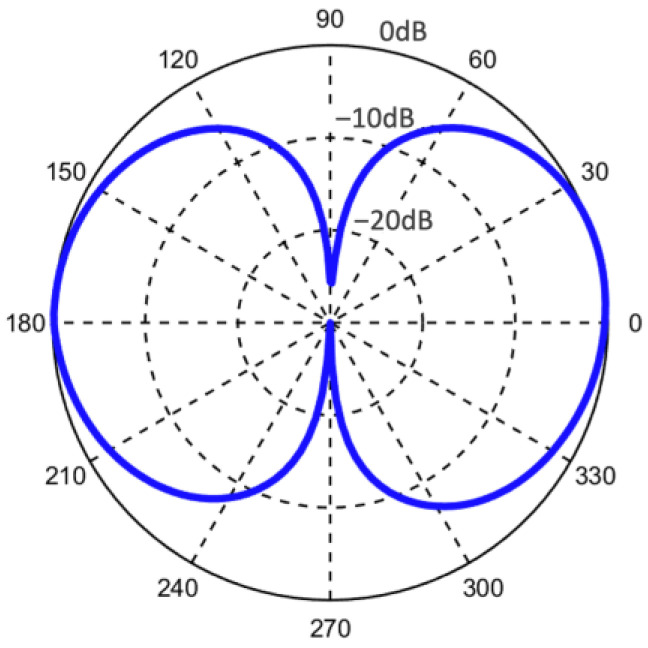
Measured directional response of the sensor mounted in the housing. The measurement was performed in an anechoic chamber showing a dipole pattern similar to that obtained using the shaker table measurement (Figure 4b).

The directional response of the sensor with housing was performed in air at the resonance frequency (1.6 kHz) by rotating the turntable; results are plotted in Figure 6. The directional response shows a cosine directional response consistent with the vibration measurements performed using a shaker (Figure 4b). Cross-axial sensitivities of −30 dB and −27 dB were measured, indicating a small asymmetry in the directional response.

### 3.3. Underwater Measurements

**Figure 7:**
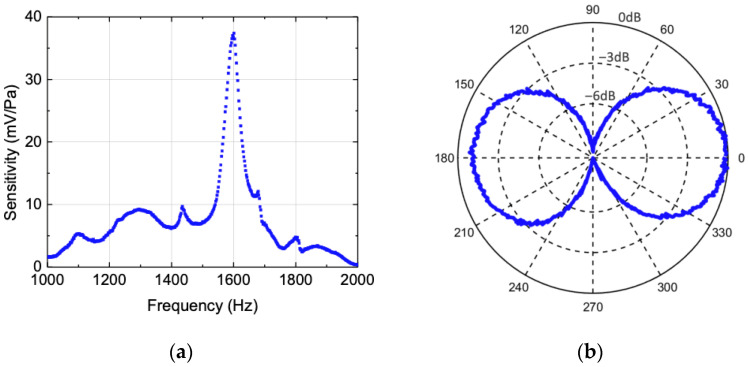
(**a**) Underwater frequency response of the sensor. The data were normalized by measuring the sound pressure using a reference hydrophone. (**b**) Directional response of the sensor measured underwater showing dipole pattern with cross-axial sensitivity of −10 dB.

The measured sensitivity as a function of frequency for the MEMS sensor is shown in Figure 7a. The data show a clear resonance at 1.6 kHz with a peak sensitivity of about 37 mV/Pa or −149 dB re 1 V/μPa. The peak position agrees well with that measured in air as shown in Figure 5b. This is expected since the sensor’s housing is filled with air, therefore the sensor, inside the housing, is not directly in contact with the surrounding water. Thus, there is no mass-loading and the damping is primarily due to the interaction of moving parts of the sensor with air inside the housing, providing characteristics similar to that observed in air. The additional peaks near 1150 Hz, 1450 Hz, 1670 Hz, and 1800 Hz visible in Figure 7a, which are different from the air measurements (Figure 5b), are probably due to structural resonances generated by the housing of the sensor when operated in underwater. We envision that this effect can be compensated for in the application end in two ways: (a) since the sensor is meant to operate near resonance, these extra peaks will be filtered out by the readout electronics; (b) if broader band operation is required, the entire frequency response of the sensor in the band of operation will be used to generate a calibration curve to take into account the presence of these peaks. The peak sensitivity was 37 mV/Pa, which indicates that a stimulus of 1 Pa at 1.6 kHz is accelerating the entire assembly to 0.0041 g.

The directional response of the sensor at resonance was carried out underwater using a rotator. The results, shown in Figure 7b, exhibit a dipole pattern, similar to those obtained in air. The jitter in the data comes from the vibrations of the rotator used in the measurement. The presented data were not mathematically smoothed. The two maxima occurred when sound is incident directly onto the front and back (0° and 180°) of the sensor, while the two minima occurred at ± 90 degrees (or when the sound arrives parallel to the plane of the sensor), as expected. It can be seen in Figure 7b that the two maxima are slightly different (less than 10%), which may be due to an asymmetry of the housing that can induce rotating motion, as opposed to pure translation, following the direction of sound. The cross-axial sensitivity was found to be −10 dB, which is significantly less than that measured in air. This is due to multiple sound reflections on the walls of the testing tank, causing an omnidirectional offset of approximately 20 dB.

**Figure 8:**
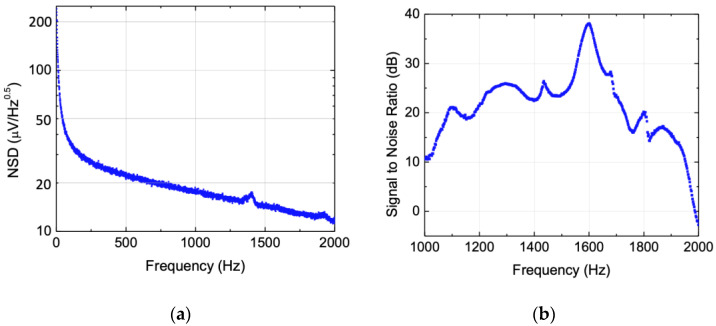
(**a**) Noise spectral density of the MEMS sensor measured underwater. (**b**) Estimated signal-to-noise ratio over 1 kHz band for an acoustic stimulus of 1 Pa (details in the text).

Noise spectral density (NSD) of the sensor was measured using a MFLI Zurich lock-in amplifier for frequencies less than 2 kHz. This range was chosen because the readout electronics were configured to filter frequencies beyond 2 kHz. Figure 8a shows the measured NSD after averaging 100 times. Note that the low frequency region is dominated by 1/f noise and the nearly constant white noise prevails beyond the 1/f noise region. The absence of resonance in the noise measurements reveals that the electronic readout is the dominant source of noise. The signal-to-noise ratio was computed for the frequency band between 1 and 2 kHz. The signal power at 1 Pa (the square of the curve shown in Figure 7) was divided by the noise power, calculated by integrating the NSD over the entire 1–2 kHz band (0.21 × 10^−6^ V^2^). The result is shown in Figure 8b where at resonance a SNR around 38 dB was achieved.

## 4. Discussion

An *Orima*-inspired MEMS acoustic sensor [26] was demonstrated in a near-neutrally buoyant configuration. One of the interesting characteristics of this type of sensor is the ability to place, by design, the resonance at lower frequencies, while preserving a relatively small form factor (~4 mm). Shaker table measurements without the sensor housing showed that, as the tilt angle was increased, the output voltage followed a cosine dependence on the vibration angle, which is similar to the directional response observed when the sensor was stimulated acoustically (Figure 6). The peak acceleration sensitivity was found to be near 5 V/g, which is comparable to accelerometers five times larger [5]. Next, the sensor was housed in a sealed air cavity and its sensitivity measured inside an anechoic chamber. The measured sensitivity at the resonance frequency (1.6 kHz) was about 95 mV/Pa. Comparison between measurements performed with the sensor unhoused and housed showed that acceleration was measured rather than sound pressure, which is desirable for underwater operation in the configuration discussed here. Underwater measurements were then carried out in a water tank using the same housing and mounting frame used for the air measurements. The frequency response remained largely the same as that obtained in air, while the maximum sensitivity was found to be about 37 mV/Pa (−149 dB re 1 V/µPa) at the resonance frequency (1.6 Hz). The effects of the temperature difference between the air and underwater characterizations were not observed since the bending resonance did not change. The temperature difference, 5 °C, was not enough to change the Young’s modulus of silicon (sensor structure) to a noticeable extent [27]. This effect might be noticeable when the sensor is operated in much colder waters.

Operating the sensor at resonance brings the advantage of significantly increasing the signal-to-noise ratio. This is possible because the noise floor is determined by the electronic readout; therefore, the increase in the SNR is directedly proportional to the increase in mechanical sensitivity. For applications that only require narrow band detection the sensor demonstrated herein can be operated with sensitivity around two to three orders of magnitude higher than the sensors reported in [17,18,19,20]. The sensor in underwater operation showed a cosine dependence on the angle of sound incidence similar to that obtained in air. For applications where direction of arrival determination is required a combination of two dipoles and one omnidirectional sensor can provide unambiguous 360-degree coverage [28]. For such applications, very small form factor vector sensors can be constructed. The noise characteristics of the sensor were also measured and the estimated signal-to-noise ratio for the sensor operated near resonance was found to be about 38 dB for a signal generated by a 1 Pa sound pressure and 1 kHz bandwidth. Since the noise is predominantly determined by the electronic readout, much improvement can be obtained. Dedicated microelectronic circuits can be designed using charge amplifiers to directly convert the alternate capacitance from the sensor’s comb fingers into voltage [29]. In this case, the sensitivity can be controlled by the readout gain. Conclusively, the results reported here indicate that MEMS directional acoustic sensors can be operated in a near-neutrally buoyant configuration for underwater applications with good directionality and high signal-to-noise ratio.

## References

[1] Leslie C.B., Kendall J.M., Jones J.L. (1956). Hydrophone for Measuring Particle Velocity. J. Acoust. Soc. Am..

[2] Moffett M.B., Trivett D.H., Klippel P.J., Baird P.D. (1998). A Piezoelectric, Flexural-Disk, Neutrally Buoyant, Underwater Accelerometer. IEEE Trans. Ultrason. Ferroelectr. Freq. Control.

[3] Agarwal A., Kumar A., Aggarwal M., Bahl R. Design and Experimentation with Acoustic Vector Sensors. Proceedings of the 2009 International Symposium on Ocean Electronics (SYMPOL 2009).

[4] Gabrielson T.B., Gardner D.L., Garrett S.L. (1995). A Simple Neutrally Buoyant Sensor for Direct Measurement of Particle Velocity and Intensity in Water. J. Acoust. Soc. Am..

[5] Zhang H., Chen H.-J., Wang W.-Z. (2014). An Underwater Acoustic Vector Sensor with High Sensitivity and Broad Band. Sens. Transducers.

[6] McConnell J.A. Practical Experiences with Inertial Type Underwater Acoustic Intensity Probes. Proceedings of the OCEANS’02 MTS/IEEE.

[7] Banner A. (1973). Simple Velocity Hydrophones for Bioacoustic Application. J. Acoust. Soc. Am..

[8] Keller B.D. (1977). Gradient Hydrophone Flow Noise. J. Acoust. Soc. Am..

[9] Gardner D., Hofler T., Baker S., Yarber R., Garrett S. (1987). A Fiber-Optic Interferometric Seismometer. J. Light. Technol..

[10] D’ Spain G.L., Hodgkiss W.S., Edmonds G.L. (1991). The Simultaneous Measurement of Infrasonic Acoustic Particle Velocity and Acoustic Pressure in the Ocean by Freely Drifting Swallow Floats. IEEE J. Ocean. Eng..

[11] Berliner M.J., Lindberg J.F. (1996). Acoustic Particle Velocity Sensors: Design, Performance, and Applications Proceedings.

[12] Franklin J.B., Barry P.J. (1996). Acoustic Particle Acceleration Sensors. AIP Conf. Proc..

[13] Cray B.A., Christman R.A. (1996). Acoustic and Vibration Performance Evaluations of a Velocity Sensing Hull Array. AIP Conf. Proc..

[14] Gray M., Rogers P.H., Zeddies D.G. (2016). Acoustic Particle Motion Measurement for Bioacousticians: Principles and Pitfalls. Proc. Meet. Acoust. 4ENAL.

[15] Josserand M.A., Maerfeld C. (1985). PVF2 Velocity Hydrophones. J. Acoust. Soc. Am..

[16] Rockstad H.K., Kenny T.W., Kelly P.J., Gabrielson T.B. (1996). A Micro-Fabricated Electron-Tunneling Accelerometer as a Directional Acoustic Sensor. Proceedings of the Acoustic Particle Velocity Sensors: Design, Performance and Applications 9/95.

[17] Roh Y., Pyo S., Lee S. (2018). Design of an Accelerometer to Maximize the Performance of Vector Hydrophones. Proceedings of the Nano-, Bio-, Info-Tech Sensors, and 3D Systems II.

[18] Guan L., Zhang G., Xu J., Xue C., Zhang W., Xiong J. (2012). Design of T-Shape Vector Hydrophone Based on MEMS. Sens. Actuators Phys..

[19] Xue C., Tong Z., Zhang B., Zhang W. (2008). A Novel Vector Hydrophone Based on the Piezoresistive Effect of Resonant Tunneling Diode. IEEE Sens. J..

[20] Edalafar F., Azimi S., Qureshi A.Q.A., Yaghootkar B., Keast A., Friedrich W., Leung A.M., Bahreyni B. (2017). A Wideband, Low-Noise Accelerometer for Sonar Wave Detection. IEEE Sens. J..

[21] Miles R.N., Robert D., Hoy R.R. (1995). Mechanically Coupled Ears for Directional Hearing in the Parasitoid Fly Ormia Ochracea. J. Acoust. Soc. Am..

[22] Rahaman A., Kim B. (2020). Sound source localization by Ormia ochracea inspired low-noise piezoelectric MEMS direcional microphone. Sci. Rep..

[23] Wang R., Shen W., Zhang W., Song J., Li N., Liu M., Zhang G., Xue C., Zhang W. (2021). Design and implementation of a jellyfish otolith-inspired MEMS vector sensor hydrophone for low frequency detection. Microsyst. Nanoeng..

[24] Touse M., Sinibaldi J., Simsek K., Catterlin J., Harrison S., Karunasiri G. (2010). Fabrication of a Microelectromechanical Directional Sound Sensor with Electronic Readout Using Comb Fingers. Appl. Phys. Lett..

[25] Yu H.J., Ding Z.Q., He X.P., Du L.M., Qu H., Zhou W., Peng B. (2015). The Research on MEMS Micro Capacitance Sensor Detection Based on MS3110. Key Engineering Materials.

[26] Wilmott D., Alves F., Karunasiri G. (2016). Bio-Inspired Miniature Direction Finding Acoustic Sensor. Sci. Rep..

[27] Vanhellemont J., Swarnakar A.K., Van der Biest O. (2014). Temperature dependent Young’s modulus of Si and Ge. ECS Trans..

[28] Davies S. Bearing Accuracies for Arctan Processing of Crossed Dipole Arrays. Proceedings of the OCEANS’87.

[29] Rabelo R., Alves F., Karunasiri G. (2019). MEMS Directional Acoustic Sensor with Charge Amplifier Based Electronic Readout. J. Acoust. Soc. Am..

